# Correlation of interleukin-6 and C-reactive protein levels in plasma with the stage and differentiation of colorectal cancer: A cross-sectional study in East Indonesia

**DOI:** 10.1016/j.amsu.2021.01.013

**Published:** 2021-01-23

**Authors:** Ferdian Hidayat, Ibrahim Labeda, Samuel Sampetoding, Ilham Jaya Pattelongi, Ronald Erasio Lusikooy, Warsinggih, M. Iwan Dani, Mappincara, M. Ihwan Kusuma, Julianus Aboyaman Uwuratuw, Erwin Syarifuddin, Muhammad Faruk

**Affiliations:** a Division of Digestive, Department of Surgery, Faculty of Medicine, Hasanuddin University, Makassar, Indonesia; b Faculty of Medicine, Khairun University, Ternate, Indonesia; c Department of Biostatistics, Faculty of Public Health, Hasanuddin University, Makassar, Indonesia; d Department of Surgery, Faculty of Medicine, Hasanuddin University, Makassar, Indonesia

**Keywords:** Colorectal cancer, Interleukin-6, C-reactive protein, Stage, Grade of differentiation

## Abstract

**Introduction:**

Tumors most often develop due to inflammatory factors, including inflammatory cells that produce cytokines and cytotoxic mediators that can stimulate malignant transformation. Knowing that interleukin-6 (IL-6) and C-reactive protein (CRP) factor into the development of colorectal cancer (CRC), we aimed to assess IL-6 and CRP's relationship with the stage and differentiation of CRC.

**Methods:**

In a sample of 46 patients with CRC, as confirmed by histopathological examination, plasma levels of IL-6 and CRP were measured from peripheral venous blood samples before surgery and examined using enzyme-linked immunosorbent assay.

**Results:**

Most patients were male (63.0%) and at least 50 years old (73.9%). A positive correlation emerged between stage of CRC and both plasma IL-6 (*r =* 0.396, *p =* .003) and CRP (*r =* 0.376, *p =* .005) levels, which the Kruskal–Wallis test indicated were highest in stage IV (IL-6: median = 25.80, *p =* .019; CRP: median = 34.10, *p =* .040). Plasma IL-6 levels (median = 25.80, *p =* .019) were higher in well-differentiated CRC, whereas plasma CRP levels (median = 34.10, *p =* .040] were higher in poorly differentiated tissue. Linear plotting revealed a linear relationship between plasma IL-6 and plasma CRP levels in patients with CRC.

**Conclusion:**

Because the stage of CRC significantly correlates with plasma IL-6 and CRP levels, IL-6 and CRP can serve as diagnostic factors in assessing the progress and prognosis of CRC.

## Highlights

• Interleukin-6 (IL-6) and C-reactive protein (CRP) levels can indicate inflammation.
• Chronic inflammation affects the progression of colorectal cancer (CRC).
• An association exists between IL-6, CRP, and stage of CRC.
• IL-6 and CRP can be diagnostic factors in assessing CRC's progression.

## 1 Introduction

Colorectal cancer (CRC) ranks among the most common types of cancer in Western countries. In the United States, for example, more than 130,000 new cases of CRC and 49,000 deaths were registered in 2016 [1]. In Indonesia, GLOBOCAN data show that in 2018, of all types of cancer, CRC had an incidence of 12.8 per 100,000 cases of cancer [[2], [3], [4]]. Location aside, epidemiological studies have shown that inflammatory bowel disease is a strong risk factor for CRC, because proinflammatory cytokines released from the immune system invade the microenvironment of the intestine and control the initiation and growth of tumors [5,6].

Intensive research on inflammation's role in CRC's initiation and growth has also recently focused on interleukin-6 (IL-6), a type of hematopoietic-mediated inflammatory cytokine responsible for activating lymphocytes. To date, although studies have demonstrated IL-6's role in mediating the progression of tumors, its contribution to the pathogenesis of chronic inflammatory diseases and cancer remains incompletely understood [7,8]. Against that trend, levels of C-reactive protein (CRP), another protein active in CRC's development, may shed light on inflammation's relationship with colorectal carcinogenesis [9]. CRP is an acute-phase protein synthesized by hepatocytes in response to IL-6, which stimulates cytokines following the inflammatory process of immune response [10]. Because CRP is associated with various immunological response mechanisms that are themselves associated with CRC's development, CRP is believed to play a significant role in its progression as well.

In our study, conducted at a referral hospital in East Indonesia, we assessed the relationship between plasma IL-6 and CRP levels with the stage and differentiation of CRC cells as important information for the early detection and diagnosis of CRC, its management as a disease, and, in turn, patients’ life expectancy.

## 2 Methods

This cross-sectional study, conducted at Dr. Wahidin Sudirohusodo Hospital in Makassar, Indonesia, followed a protocol approved by our institution's ethics committee (registration no.: 515/UN4.6.4.5.31/PP36/2020) and has been registered with the Research Registry (no. 6414). Herein, we report our work in accordance with the criteria of Strengthening the Reporting of Cohort Studies in Surgery [11].

### 2.1 Population and sample

We formed a sample of 46 patients—29 males and 17 females, aged 54.4 years on average (range: 21–72 years)—who needed surgical treatment due to CRC, the presence of which was confirmed by histopathological examination with a rectal biopsy or colonoscopy prior to biopsy. To be eligible to participate, patients had to have a diagnosis of CRC based on a histopathological examination; demonstrate normal liver function, kidney function, urinalysis, and chest radiographs; have no history of hemostatic disorders; not currently suffer from acute or chronic infectious diseases as evidenced by routine blood results and consent to participate. Exclusion criteria were damaged blood samples or malignancy in another organ (i.e., synchronous tumor). Stages of CRC were determined with reference to the American Joint Committee on Cancer's 2017 TNM staging system [12], whereas grades of differentiation were determined based on the grading of the World Health Organization [6,13].

### 2.2 Sample examination

Prior to surgery, 3 mL of peripheral venous blood was collected from each patient in a Vacutainer test tube and immediately transported to the laboratory. After spontaneous precipitation for 20 min, each blood sample was centrifuged at 3000 rpm for 10 min, and the serum was separated into two aliquots. One aliquot was stored at −80 °C for the later analysis of IL-6 serum concentration, namely with an enzyme-linked immunosorbent assay (ELISA) kit (catalog no. E0090Hu) and reagents from the Bioassay Technology Laboratory (Shanghai, China) according to the manufacturer's instructions [4,14]. The other aliquot was tested for CRP serum level on the day of sampling, namely with an ELISA kit (reference no. CAN-CRP-4360) from Diagnostics Biochem Canada Inc. (Ontario, Canada) according to the manufacturer's instructions [[15], [16], [17]]. Measurements of IL-6 concentration were expressed in picograms per milliliter (pg/mL) and of CRP serum level in milligrams per liter (mg/L).

### 2.3 Statistical analysis

Data were divided based on type and presented as graphs and tables. The Statistical Package for the Social Sciences for Windows (version 23.0; IBM Corp, Armonk, NY) was used to determine how IL-6 and CRP levels related to the stage and grade of differentiation. Data were analyzed using the Kruskal–Wallis test plus Mann–Whitney test to assess the difference in median levels of IL-6 and CRP at each stage and differentiation of CRC. Spearman's rank correlation test was administered to assess the correlation between variable levels of serum IL-6 and CRP with the stage and differentiation of CRC. Last, data were analyzed with a one-way ANOVA, followed by a multiple comparisons test using least significance difference (LSD). All *p* values less than .05 were considered to be statistically significant.

## 3 Results

### 3.1 Sample characteristics

Data analysis was conducted on 46 patients with CRC, all 21–72 years of age, most of whom were male (63.0%) and at least 50 years old (73.9%). As shown in Table 1, plasma IL-6 levels were 0.5–62.1 pg/mL (*Mean* = 15.27 ± 15.01), while plasma CRP levels were 2.4–71.4 mg/L (*M* = 21.70 ± 17.74).

**Table 1:** Descriptive statistics of the sample by age, IL-6 level, and CRP level (N = 46).

Variable	Min.	Max.	Mean	SD
Age (years)	21	72	54.41	11.09
IL-6 (pg/mL)	0.5	62.1	15.27	15.01
CRP (mg/L)	2.4	71.4	21.70	17.74

In the sample, the greatest number of participants had CRC in stage III (45.7%), while the most common degree of cell differentiation was good (47.8%), as detailed in Table 2.

**Table 2:** Distribution of sex, age, stage of CRC, and grade of differentiation in the sample (*N* = 46).

Variable		n	%
Sex	Male	29	63.0
	Female	17	37.0
Age	<50 years	12	26.1
	≥50 years	34	73.9
Stage	II	12	26.0
	III	21	45.7
	IV	13	28.3
Grade of differentiation	Good	13	28.3
	Moderate	22	47.8
	Poor	11	23.9

### 3.2 Relationship between stage of CRC and plasma IL-6 and CRP levels

Data analysis revealed a positive correlation between plasma IL-6 levels and stage of CRC, with a Spearman's correlation coefficient of *r =* 0.396 (*p =* .003). Results of the Kruskal–Wallis difference test also showed that plasma IL-6 levels among patients with stage IV (median = 25.80) were higher than those among patients with stage III (8.40) and stage II (4.45) of the disease (*p =* .019), as shown in Table 3.

**Table 3:** Relationship between plasma IL-6 levels and stage of CRC.

Plasma IL-6 levels (pg/mL)		Stage			*p*
		II (*n* = 12)	III (*n* = 21)	IV (*n* = 13)	
Comparative test	Median (Min.–max.)	4.45 (0.5–36.6)	8.40 (1.4–35.9)	25.80 (3.5–62.1)	.019^a^
Correlation test	Spearman's correlation coefficient (*r =* .396)				.003

a Kruskal–Wallis test plus Mann–Whitney test.

**Fig. 1:**
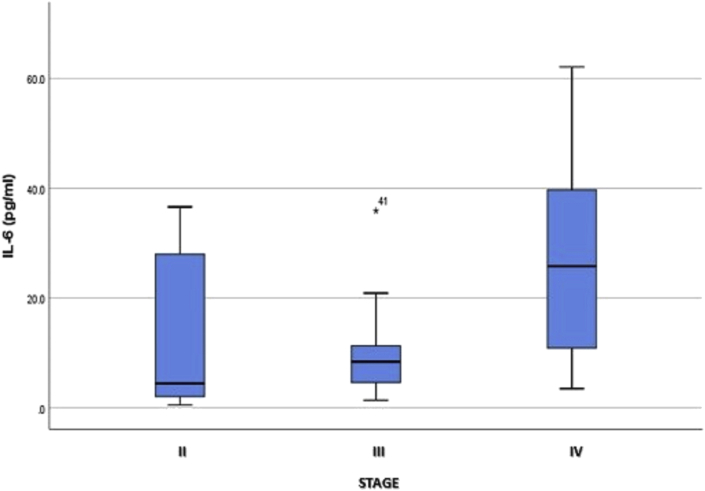
Box plot of plasma IL-6 levels at each stage of CRC.

The box plot in Fig. 1 shows that although plasma IL-6 levels at each stage of CRC were not normally distributed, mean plasma IL-6 levels were far higher in stage IV than in stages III and II. By comparison, a positive correlation emerged between plasma CRP levels and stage of CRC, according to Spearman's correlation coefficient (*r =* .376, *p =* .005). The results of the Kruskal–Wallis difference test also revealed that plasma CRP levels among patients with stage IV (median = 34.10) exceeded those among patients with stage III (16.00) and stage II (7.75) of the disease (*p =* .040), as shown in Table 4.

**Table 4:** Relationship between plasma CRP levels and stage of CRC.

Plasma CRP levels (mg/L)		Stage			*P*
		II (*n* = 12)	III (*n* = 21)	IV (*n* = 13)	
Comparative test	Median (Min.–max.)	7.75 (2.8–56.7)	16.00 (2.5–39.7)	34.10 (2.4–71.4)	.040^a^
Correlation test	Spearman's correlation coefficient (*r =* .376)				.005

a One-way ANOVA plus LSD.

**Fig. 2:**
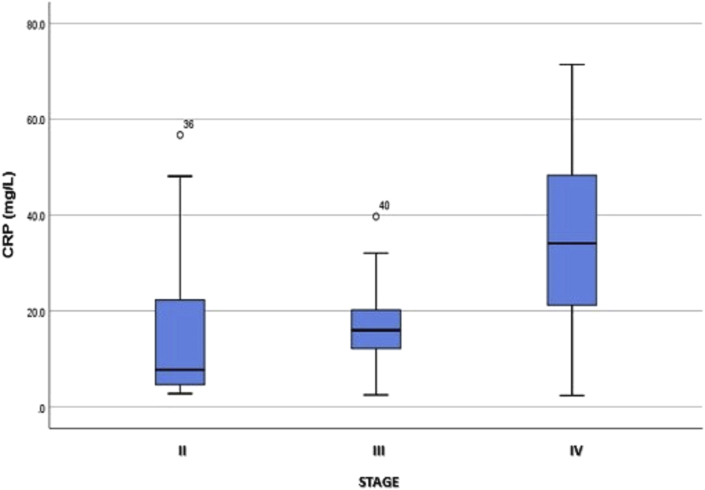
Box plot of plasma CRP levels at each stage of CRC.

The box plot in Fig. 2 shows that although plasma CRP levels at each stage were not normally distributed, mean plasma CRP levels were far higher among patients with CRC at stage IV than stage II or III.

### 3.3 Relationship between grade of differentiation and plasma IL-6 and CRP levels

**Fig. 3:**
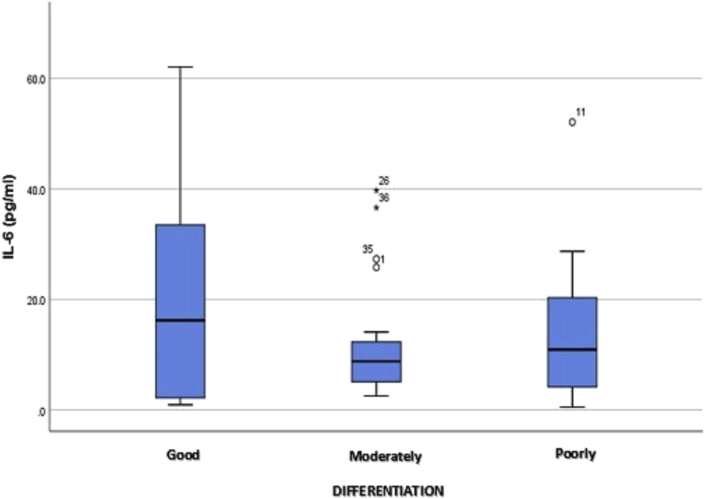
Box plot of plasma IL-6 levels at each grade of CRC differentiation.

Data analysis (Table 5) revealed no correlation between plasma IL-6 levels and the grade of differentiation among patients with CRC, with a Spearman's correlation coefficient of *r =* −0.044 (*p =* .384). According to a comparative data analysis, the highest increase in plasma IL-6 levels was with good differentiation (16.20, *p =* .819), followed by poor and moderate differentiation. Those results indicate no significant relationship between plasma IL-6 levels and the grade of CRC differentiation. The box plot in Fig. 3 shows that plasma IL-6 levels at each stage were not normally distributed and that the mean level was highest in well-differentiated CRC.

**Table 5:** Relationship between plasma IL-6 levels and grade of differentiation in CRC.

Plasma IL-6 level (pg/mL)		Grade of differentiation			*p*
		Good (*n =* 13)	Moderate (*n =* 22)	Poor (*n =* 11)	
Comparative study	Median (Min.–max.)	16.20 (0.9–62.1)	8.80 (2.5–39.7)	10.90 (0.5–52.1)	.819^a^
Correlation study	Spearman's correlation coefficient (*r =* −.044)				.384

a Kruskal–Wallis test plus Mann–Whitney test.

With a Spearman's correlation coefficient of *r =* 0.245 (*p =* .051), no significant correlation surfaced between plasma CRP levels and the grade of differentiation. The results of the Kruskal–Wallis comparative test also showed that the plasma CRP level of patients with poorly differentiated CRC (median = 26.40) exceeded those of moderately (18.60) and well-differentiated CRC (8.90), albeit not significantly (*p >* .05), as detailed in Table 6.

**Table 6:** Relationship between plasma CRP levels and grade of differentiation in CRC.

Plasma CRP level (mg/L)		Grade of differentiation			*p*
		Good (*n =* 13)	Moderate (*n =* 22)	Poor (*n =* 11)	
Comparative study	Median (Min.–max.)	8.90 (2.8–71.4)	18.60 (2.4–56.7)	26.40 (4.1–63.8)	.401^a^
Correlation study	Spearman's correlation coefficient (*r =* .245)				.051

a One-way ANOVA plus LSD.

**Fig. 4:**
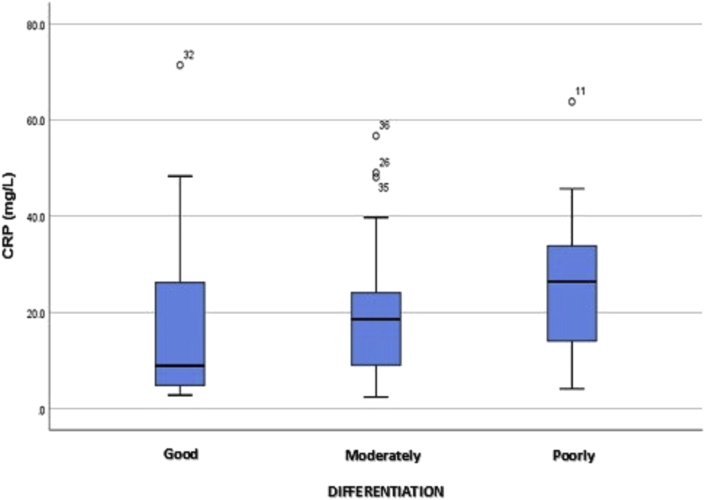
Box plot of plasma CRP levels at each grade of CRC differentiation.

The box plot in Fig. 4 shows that plasma CRP levels at each grade of differentiation were not normally distributed. Mean plasma CRP level was higher at a poorer grade of CRC differentiation, and though a linear pattern surfaced between the grade of differentiation and plasma CRP level, it was not significant.

### 3.4 Relationship between plasma IL-6 and plasma CRP levels in CRC

**Fig. 5:**
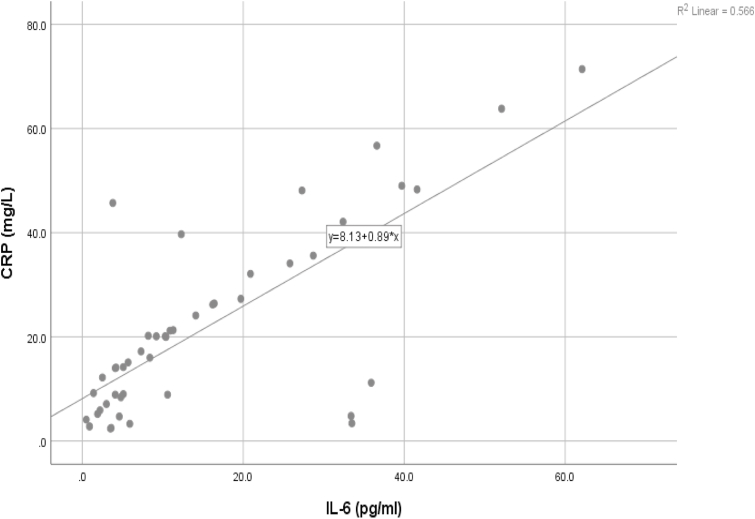
Scatter plot between plasma IL-6 and plasma CRP levels in CRC.

To determine the relationship between plasma IL-6 and plasma CRP levels in patients with CRC, we administered Pearson's correlation test and created a scatter plot. Results showed a relationship between plasma IL-6 and plasma CRP levels with a Pearson's correlation coefficient of *r =* .752 (*p* < .001, *R*^2^ = 0.566). Fig. 5 shows the linear relationship between plasma IL-6 and plasma CRP levels in patients with CRC. In short, the greater the IL-6 level, the greater the plasma CRP level.

## 4 Discussion

Our sample of 46 patients with CRC, most of whom were male (63.0%) and at least 50 years old (73.9%), exhibited trends similar to ones observed by Kuipers (2015) and Brenner (2014), namely that CRC's incidence is highest in men and individuals more than 50 years of age. Those results may be attributed to variations in diet and lifestyle. By stage of CRC, the most common in the sample were stages III and IV, which indicates that most patients presented at the hospital well after early-stage CRC. That trend corroborates Siegel's (2017) data from Colorectal Cancer Statistics, which offer the additional insight that low socioeconomic status factors into the lack of early detection of CRC and inadequate access to health care [2].

We also observed a significant relationship between the stage of CRC and levels of IL-6 in blood plasma (*p* < .003), which peaked in patients with stage IV of the disease (62.1 pg/mL). That result aligns with Rasic's (2018) findings of a significant increase in IL-6 levels according to the stage of CRC, with values that also peaked in stage IV [5]. Along similar lines, Zeng et al. (2017), who examined the relationship between IL-6 in tissues and the risk of developing CRC, found a significant positive correlation between IL-6 levels and the stage of CRC [18].

Investigating the expression of IL-6 in CRC tissue, Jasmin et al. (2019) identified a significant relationship between IL-6 and both the stage and grade of differentiation of CRC, particularly that IL-6 contributes to the promotion and proliferation of tumors. Those results suggest a strong association between plasma IL-6 levels and CRC's development, as well as IL-6's significant predictive value in assessing stages II–IV of CRC given its association with chronic inflammatory processes and significant role in CRC's genesis. Taken together, those biomarkers could offer a useful tool for monitoring CRC's progression [19]. Two other studies have shown that the higher the stage of CRC, the higher the level of IL-6 in blood plasma. Such results confirm a relationship between chronic inflammation and cancer's progression, in which IL-6 ranks among the cytokines active in the inflammatory response. At least two studies have added that IL-6 closely relates to all stages of tumorigenesis, including cellular transformation, promotion, survival, proliferation, invasion, angiogenesis, and metastasis [20,21].

Also mirroring past results, our study revealed a significant relationship between the stage of CRC and CRP levels in blood plasma (*p* < .003), which peaked in stage IV of the disease. In particular, Kersten et al. (2013) found that CRP levels relate significantly to the stage and prognosis of CRC [22].

Because CRP is an acute-phase protein that enters the circulation in response to tissue damage and inflammation and can be a biological marker of chronic systemic inflammation, moderate increases in CRP levels are associated with an increased risk of CRC [5,[21], [22], [23], [24], [25]].

We did not find a significant relationship between the grade of differentiation of CRC and plasma IL-6 levels (*p >* .05), the highest median of which occurred in well-differentiated CRC (16.20 pg/mL). That result confirms Zeng's (2017) finding of no significant relationship between IL-6 levels and the grade of CRC's differentiation [18]. We also found no significant relationship between the grade of differentiation and CRP levels in plasma (*p >* .05). Even though the highest median level of CRP occurred in poorly differentiated CRC (26.40 mg/L), a linear but non-significant pattern surfaced between the grade of differentiation and plasma CRP levels. Contrary to our results, Kersten et al. (2013) found that CRP levels significantly related to CRC's grade of differentiation, possibly due to the small sample, even despite the linear pattern between them [20]. Those results suggest a relationship between inflammation and CRC, in which IL-6 and CRP play important roles not only as proinflammatory cytokines but also as contributors to CRC's development [22].

## 5 Conclusion

Our findings showcase an association between levels of both IL-6 and CRP in blood plasma and the stage of CRC but no significant relationship between those levels and the grade of CRC's differentiation. The relationship revealed between both IL-6 and CRP levels and the stage of the disease may facilitate the diagnosis of early-stage CRC and thus improve its management and the life expectancy of patients with the disease. Combined, the IL-6 and CRP biomarkers can also serve as diagnostic aids in assessing the progression and prognosis of CRC.

Among our study's limitations were the small sample and the brief study period. Above all, the sample was unevenly distributed due to lacking patients with CRC in stage I, who rarely present at referral hospitals such as ours. In the future, researchers should undertake longitudinal studies with longer study periods in order to assess IL-6 and CRP's relationship with CRC.

## Provenance and peer review

Our study was non-commissioned and externally peer-reviewed.

## Declaration of competing interest

Ferdian Hidayat, Ibrahim Labeda, Samuel Sampetoding, Ilham Jaya Pattelongi, Ronald Erasio Lusikooy, Warsinggih, M. Iwan Dani, Mappincara, M. Ihwan Kusuma, Julianus A. Uwuratuw, Erwin Syarifuddin, and Muhammad Faruk declare that they have no conflict of interest.

## Registration of research studies

This cross-sectional study has been registered with the Research Registry (no. 6414).

https://www.researchregistry.com/browse-the-registry#home/registrationdetails/5ff166088bd9cd001b0f2ae5/
